# Asynchronous Versus Synchronous Screening for Depression and Suicidality in a Primary Health Care System: Quality Improvement Study

**DOI:** 10.2196/50192

**Published:** 2024-05-01

**Authors:** Amelia Sattler, Julia Dunn, Marleni Albarran, Charlotte Berger, Ana Calugar, John Carper, Lalitha Chirravuri, Nadine Jawad, Mira Zein, Mark McGovern

**Affiliations:** 1 Department of Medicine, Stanford University School of Medicine, Palo Alto, CA, United States; 2 Department of Psychiatry and Behavioral Sciences, Stanford University School of Medicine, Palo Alto, CA, United States; 3 Technology and Digital Solutions, Stanford Health Care, Palo Alto, CA, United States; 4 Department of Quality, Stanford Health Care, Stanford, CA, United States; 5 University Healthcare Alliance, Stanford, CA, United States; 6 Stanford University School of Medicine, Palo Alto, CA, United States

**Keywords:** depression diagnosis, primary health care methods, electronic health records utilization, quality improvement, web-based universal screening methods, suicide prevention and control, screening, depression, asynchronous, synchronous, primary care, suicide, intervention, prevention

## Abstract

**Background:**

Despite being a debilitating, costly, and potentially life-threatening condition, depression is often underdiagnosed and undertreated. Previsit Patient Health Questionnaire-9 (PHQ-9) may help primary care health systems identify symptoms of severe depression and prevent suicide through early intervention. Little is known about the impact of previsit web-based PHQ-9 on patient care and safety.

**Objective:**

We aimed to investigate differences among patient characteristics and provider clinical responses for patients who complete a web-based (asynchronous) versus in-clinic (synchronous) PHQ-9.

**Methods:**

This quality improvement study was conducted at 33 clinic sites across 2 health systems in Northern California from November 1, 2020, to May 31, 2021, and evaluated 1683 (0.9% of total PHQs completed) records of patients endorsing thoughts that they would be better off dead or of self-harm (question 9 in the PHQ-9) following the implementation of a depression screening program that included automated electronic previsit PHQ-9 distribution. Patient demographics and providers’ clinical response (suicide risk assessment, triage nurse connection, medication management, electronic consultation with psychiatrist, and referral to social worker or psychiatrist) were compared for patients with asynchronous versus synchronous PHQ-9 completion.

**Results:**

Of the 1683 patients (female: n=1071, 63.7%; non-Hispanic: n=1293, 76.8%; White: n=831, 49.4%), Hispanic and Latino patients were 40% less likely to complete a PHQ-9 asynchronously (odds ratio [OR] 0.6, 95% CI 0.45-0.8; *P*<.001). Patients with Medicare insurance were 36% (OR 0.64, 95% CI 0.51-0.79) less likely to complete a PHQ-9 asynchronously than patients with private insurance. Those with moderate to severe depression were 1.61 times more likely (95% CI 1.21-2.15; *P*=.001) to complete a PHQ-9 asynchronously than those with no or mild symptoms. Patients who completed a PHQ-9 asynchronously were twice as likely to complete a Columbia-Suicide Severity Rating Scale (OR 2.41, 95% CI 1.89-3.06; *P*<.001) and 77% less likely to receive a referral to psychiatry (OR 0.23, 95% CI 0.16-0.34; *P*<.001). Those who endorsed question 9 “more than half the days” (OR 1.62, 95% CI 1.06-2.48) and “nearly every day” (OR 2.38, 95% CI 1.38-4.12) were more likely to receive a referral to psychiatry than those who endorsed question 9 “several days” (*P*=.002).

**Conclusions:**

Shifting depression screening from in-clinic to previsit led to a dramatic increase in PHQ-9 completion without sacrificing patient safety. Asynchronous PHQ-9 can decrease workload on frontline clinical team members, increase patient self-reporting, and elicit more intentional clinical responses from providers. Observed disparities will inform future improvement efforts.

## Introduction

In 2020, approximately 21 million adults in the United States experienced at least 1 major depressive episode, with only 66% receiving treatment, and nearly 46,000 people dying from suicide [1,2]. Primary care health systems can help prevent suicide by effectively screening and connecting patients with early intervention and treatment for depression [3,4].

The relationship between depression and the risk of suicide is well established and highlights the urgency of proactive intervention [5]. Depression is the most common psychiatric disorder in people who die by suicide [6]. A majority of individuals who die by suicide visited their primary care provider in the year preceding their death [7-9]. Thus, primary care is a pivotal setting for identifying suicide risk and initiating mental health care, starting with the implementation of effective screening processes [3,4].

Despite guidelines and recommendations for preventive annual depression screening that includes suicide risk assessment, health systems face a dilemma in integrating these measures into routine care [10-12]. Little evidence exists on the predictors and outcomes associated with asynchronous depression screening programs, such as which patient groups are more likely to engage in asynchronous depression screening and whether asynchronous screening affects a provider’s clinical response [13-15]. Asynchronous previsit patient questionnaires can ease the administrative burden on frontline clinical staff and may help to efficiently identify problems ahead of primary care visits. However, concerns exist regarding the need for an immediate clinical response and the potential liability associated with patient responses indicating a high risk of severe depression that may result in self-harm or suicide. This concern results in health systems compromising by administering incomplete asynchronous depression screening that excludes questions overtly asking patients to report thoughts of self-harm or wishing they were dead. One resolution to this dilemma was the development of the Patient Health Questionnaire-8 (the 8-item version). This measure is an adaptation of the more widely adopted and validated Patient Health Questionnaire-9 (PHQ-9; the 9-item version) but does not include the ninth question, which is about suicidal thoughts [16].

Most depression screening and suicide risk assessments currently occur in an in-person clinical encounter, allowing for immediate evaluation, triage, and management of high-risk patients [17-19]. However, the dramatic shift from direct face-to-face patient care to remote care in the context of the COVID-19 pandemic rendered synchronous clinic-based screening programs insufficient. Meanwhile, during the COVID-19 pandemic, the prevalence of symptoms of depressive disorders nearly quadrupled [20]. This dramatic increase in depressive symptoms during the pandemic further heightened the urgency to adapt and optimize screening processes [21].

Screening for suicide risk remotely and asynchronously raises concerns about the risk of a patient harming themselves when support is not immediately available [22,23], particularly because no standards exist for immediate response to electronic screening for self-harm and suicidal intent [13]. However, there is robust evidence that patients are unlikely to attempt or die by suicide within a week after positively endorsing question 9 on a PHQ-9 [24]. Stated plainly, while question 9 is an important marker of disease severity, it has little predictive utility for acute risk of suicide [16,24,25].

During the pandemic, our primary care system redesigned our depression screening program to be effective for both remote and in-clinic visits by including both asynchronous screening ahead of visits and synchronous screening during visits for those patients who did not complete the asynchronous PHQ-9. Our overarching goal was to increase depression screening during the pandemic while decreasing the administrative burden on overwhelmed frontline clinical teams. We launched an automated electronic previsit depression screening workflow with the support of a multidisciplinary integrated behavioral health (IBH) team. These efforts align with the National Action Alliance for Suicide Prevention’s COVID-19 “guidance: screening for suicide risk during telehealth visits [22]. Our program has broad clinical implications, as it increased rates of identifying depression and suicide risk in our patients.

In this quality improvement study, we sought to describe and evaluate the differences in patient characteristics and provider responses based on asynchronous and synchronous completion of a depression screening questionnaire. Little evidence exists on the predictors and outcomes associated with remote depression screening programs [13-15]. Through this study, we aim to contribute valuable insights that can inform future strategies in suicide prevention within primary care settings.

## Methods

### Study Setting

This quality improvement study was conducted in 2 primary care health systems in Northern California from November 1, 2020, to May 31, 2021. Stanford Health Care (SHC) includes 11 clinics and cares for 60,000 patients annually. University Healthcare Alliance (UHA) comprises 32 clinics and cares for 120,000 patients annually.

### Depression Screening Program Description

**Figure 1.**
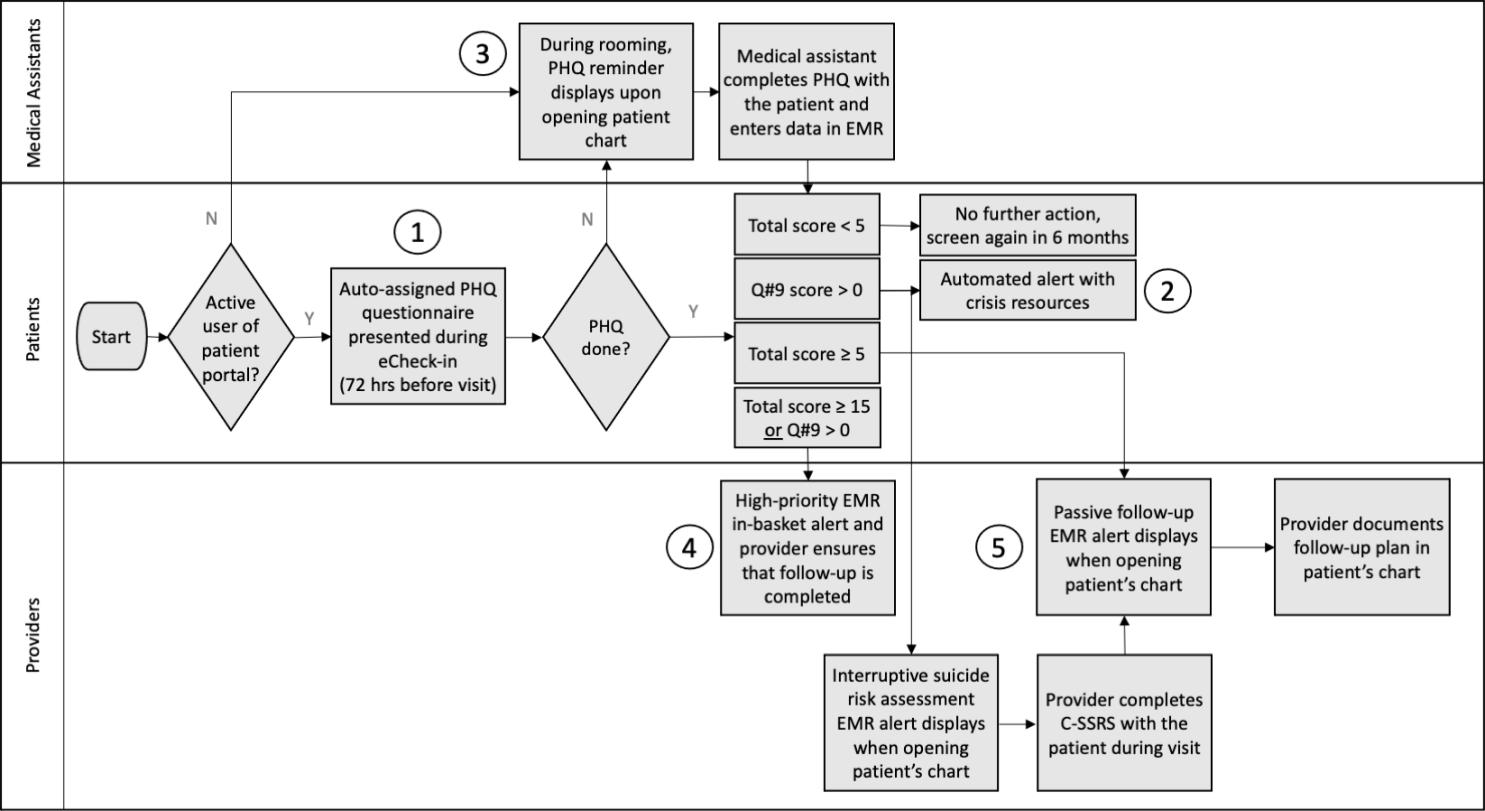
Depression screening program process map. (1) Electronic PHQ questionnaires presented to patients up to 3 days before a scheduled clinic visit, embedded into the electronic check-in procedure; (2) automated electronic patient alerts containing crisis resources displayed to patients at the time of questionnaire completion if they responded with a high-risk score; (3) reminders in the EMR prompting clinical teams to administer depression screening questionnaires during visits if patients did not complete the previsit questionnaire; (4) provider EMR alerts if a patient responded with a high-risk score; (5) reminders in the EMR prompting providers to document follow-up plans for patients who screened positive for symptoms of depression and curated decision-support tools in the EMR to assist providers with point-of-care clinical decision-making. C-SSRS: Columbia-Suicide Severity Rating Scale; EMR: electronic medical record; PHQ: Patient Health Questionnaire; Q#9: question 9 of the PHQ questionnaire (“Over the last two weeks, how often have you been bothered by thoughts that you would be better off dead or of hurting yourself”).

Our universal depression screening program was launched in 33 clinics (28 at UHA and 5 at SHC) with an automated electronic questionnaire workflow and IBH-specific clinical resources to support primary care providers managing patients who screen positive for symptoms of depression. The PHQ-9 workflow included the following elements (Figure 1): (1) electronic PHQ presented to patients up to 3 days before a scheduled visit, as part of the electronic advance check-in procedure (branching PHQ-2-to-9 for patients without an existing diagnosis of depression or a full PHQ-9 for patients with an existing diagnosis of depression); (2) an automated electronic patient alert containing crisis resources displayed to patients at the time of questionnaire completion if they responded with a high-risk score; (3) reminders in the electronic medical record (EMR) prompting clinical teams to administer PHQs synchronously during visits if patients did not complete a previsit; (4) provider EMR alerts if a patient responded with a high-risk score; and (5) reminders in the EMR prompting providers to document follow-up plans for patients with symptoms of depression and curated decision-support tools in the EMR to assist providers with point-of-care clinical decision-making. Clinical resources at SHC included a triage nurse team, IBH social workers (SWs), and a consulting psychiatrist. Additional resources, such as psychiatrists and therapists, were available in the community for patients in both health systems.

### Measures

To assess the short-term impact of the screening program, we tracked total, asynchronous, and in-clinic PHQ-9 completion rates using an EMR report. The PHQ-9 has been widely applied and validated for use in primary care settings for the screening of depression with high sensitivity (74%) and specificity (91%) [26,27]. The ninth item evaluates passive thoughts of death or self-injury and has more limited utility as a measure of suicidal risk unless paired with a validated suicide risk assessment instrument and appropriate clinical responses [28]. Specifically, question 9 reads as follows: “Over the last two weeks, how often have you been bothered by thoughts that you would be better off dead or of hurting yourself.” The response options were the following: Not at all (0), Several days (1), More than half the days (2), and Nearly every day (3). Patients with a score of 1 or higher on question 9 were included in the analysis. For this study, trained reviewers performed chart reviews of every patient who responded positively to the 9th question on a PHQ-9. Reviewers (MA and NJ) had experience navigating EMRs and were trained by an experienced clinician (AS) to identify relevant details and document them in a shared Excel worksheet.

Patient demographic characteristics were based on the EMR and included sex (male and female), race (White, Black or African American, Asian, and other/unknown), age in years, ethnicity (non-Hispanic, non-Latino and Hispanic/Latino), health insurance, encounter type (telemedicine or office visit), appointment status (completed, canceled, left, or no-show), and PHQ-9 score.

The record reviewers scanned the EMR for patient outcomes and providers’ clinical responses to evaluate patient safety and provider adherence to recommended clinical workflows. Clinical response included actions that providers took to manage or improve a patient’s depression, including prescribing new antidepressant medications; adjusting the dose of a current antidepressant medication; electronic consultation with a psychiatrist; referral to IBH SWs; referral to a psychiatry specialist; recommending ongoing management with an established behavioral health specialist outside our health system; linkage to the triage nurse for risk assessment; and completing a standardized suicide risk assessment, specifically the Columbia-Suicide Severity Rating Scale (C-SSRS). The C-SSRS is a psychometrically established and the gold standard for evaluating suicidal ideation severity and suicidal behaviors [29]. The scale contains 2 subsets of items with the first subset capturing the past-month severity of suicidal ideation and the second subset evaluating the past 3-month presence of suicide attempts.

Chart reviews for SHC patients included a review of free-text documentation in clinical notes and discrete data captured in standard reports. Chart reviews for UHA were limited to a review of discrete data captured in standard reports and focused review for any adverse patient events, such as suicide attempt or completion, that occurred between the time of questionnaire completion and the patient’s clinical encounter. For this reason, clinical response outcomes were only measured in the SHC sample. Patient safety was inferred based on whether a patient attended a subsequent clinical encounter. For patients who were deceased at the time of retrospective chart review, the cause of death was determined based on chart review and classified as “suicide” or “other,” such as terminal cancer.

### Statistical Analysis

Descriptive statistics were used to summarize participant characteristics using frequency distributions, means, and medians. PHQ-9 categories were made based on the total score and grouped based on validated cut-offs of negative (0-4), mild (≥5), moderate (≥10), moderately severe (≥15), and severe (≥20) [25]. Because of the small sample size, Native Hawaiian, Pacific Islander, and American Indian/Alaska Native were recombined with “Other.” Health insurance was categorized into Private, Medicare, Medicaid, or other/unknown. Age was categorized into groups as follows: 12-17, 18-39, 40-59, 60-79, and ≥80 years.

Cross-tabulations and univariate logistic regressions were used for the primary analyses to understand the relationship between the independent variables of various patient demographic characteristics and the dependent variable of completing the PHQ-9 asynchronously or synchronously. In a post hoc analysis, a 2-tailed *t* test was used to compare the mean PHQ-9 score among patients who completed the PHQ-9 asynchronously and synchronously.

Another series of univariate logistic regressions were fitted to evaluate for a difference in clinical response based on completing the PHQ-9 before or during a visit. In these models, we evaluated the relationships between the clinical response outcomes as the dependent variable and 2 different independent variables, including the asynchronous completion of the PHQ-9 and response to the ninth item of the PHQ-9. Bonferroni corrections were applied to mitigate type I errors. All analyses were conducted using Stata (version 17; StataCorp).

### Ethical Considerations

This project was determined not to be human subject research by the Stanford Institutional Review Board (IRB-62520) [30]. The data used for this study were deidentified.

## Results

### Descriptive Characteristics

During the study period, our program sent 202,681 PHQs to patients ahead of their clinic visit, and patients completed approximately 184,700 (91%) of them. Of the total questionnaires sent, 119,389 (58.9%) were completed asynchronously: 43,979 (65.2%) at SHC and 75,410 (55.8%) at UHA.

Of the 184,700 PHQs completed, 1683 (0.9%) patients responded with a score of 1 or higher on question 9 and were included in the sample (Table 1): 456 patients (1.0% of total patients screened) at SHC and 1227 (0.9% of total patients screened) at UHA. Nearly half completed the PHQ-9 asynchronously (n=826, 49.4%). The sample included primarily female (n=1071, 63.6%) and non-Hispanic (n=1293, 76.8%) patients, and the mean age was 46.4 (SD 21.1) years. Most of the sample consisted of White (n=831, 49.4%) or Asian (n=314, 18.7%) patients. Most had private health insurance (n=1025, 60.0%) or Medicare (n=525, 31.2%). A minority (n=71, 4.2%) had Medicaid.

**Table 1.** Descriptive characteristics comparing synchronous and asynchronous completion of the Patient Health Questionnaire-9 (PHQ-9).

Characteristics		Synchronous (n=857)	Asynchronous (n=826)	Total (n=1683)
**Sex, n (%)**				
	Male	301 (49.2)	311 (50.8)	612 (36.4)
	Female	556 (51.9)	515 (48.1)	1071 (63.6)
Age (years), mean (SD)		47.3 (22.3)	45.3 (19.9)	46.4 (21.1)
**Race, n (%)**				
	American Indian/Alaska Native	6 (66.7)	3 (33.3)	9 (0.5)
	Asian	138 (43.9)	176 (56.1)	314 (18.7)
	Black or African American	66 (60.0)	44 (40.0)	110 (6.5)
	Native Hawaiian or Pacific Islander	13 (54.2)	11 (45.8)	24 (1.4)
	White	432 (52.0)	399 (48.0)	831 (49.4)
	Other/unknown	202 (51.1)	193 (48.9)	395 (23.5)
**Ethnicity, n (%)**				
	Hispanic/Latino	139 (61.2)	88 (38.8)	227 (13.5)
	Non-Hispanic/non-Latino	623 (48.2)	670 (51.8)	1293 (76.8)
	Other/unknown	95 (58.3)	68 (41.7)	163 (9.7)
**Health insurance, n (%)**				
	Private	482 (47.0)	543 (53.0)	1025 (61.0)
	Medicare	304 (57.9)	221 (42.1)	525 (31.2)
	Medicaid	33 (46.5)	38 (53.5)	71 (4.2)
	Other/unknown	38 (61.3)	24 (38.7)	62 (3.7)
**Preferred language, n (%)**				
	English	808 (50.8)	782 (49.2)	1590 (94.5)
	Spanish	15 (65.2)	8 (34.8)	23 (1.4)
	Other	34 (48.6)	36 (51.4)	70 (4.2)
**Living status, n (%)**				
	Alive	846 (50.8)	818 (49.2)	1664 (98.9)
	Deceased	11 (57.9)	8 (42.1)	19 (1.1)
**Suicide attempt, n (%)**				
	Yes	—^a^	—	0 (0)
	No	—	—	1683 (100)
**Died by suicide, n (%)**				
	Yes	—	—	0 (0)
	No	—	—	1683 (100)
**PHQ-9 ninth item, n (%)**				
	Several days	606 (50.3)	599 (49.7)	1205 (71.6)
	More than half the days	148 (48.5)	157 (51.5)	305 (18.1)
	Nearly every day	103 (59.5)	70 (40.5)	173 (10.3)

a Not applicable.

At the time of chart review, 18 (1.1%) patients were deceased. Based on a detailed chart review, none of these patients died by suicide. No patients in the sample were documented to have attempted suicide between the time of questionnaire completion and their clinical encounter. Most patients responded to question 9 with “several days” (n=1205, 71.6%), followed by “more than half the days” (n=305, 18.1%) and “nearly every day” (n=173, 10.3%).

Regarding clinical responses, a minority of patients (n=361, 20.8%) received a new medication or dose adjustment (Table 2). A subset of patients were referred to psychiatry (n=205, 33.7%), SW (n=53, 11.5%), an external specialist (n=96, 21.1%), or a triage nurse (n=7, 1.5%). Some providers placed an eConsult to Psychiatry (n=34, 7.4%), where the provider directly communicated with the psychiatrist about a clinical question and received a response within 3 business days. None were sent to the emergency room for urgent evaluation. Most patients completed a C-SSRS during their visit (n=1176, 74.5%).

**Table 2.** Clinical response in the Stanford Health Care (SHC) and University Healthcare Alliance (UHA) samples by asynchronous and synchronous completion of the Patient Health Questionnaire-9 (PHQ-9).

Clinical response			Questionnaire completion		
			Synchronous	Asynchronous	Total
**SHC (n=456), n (%)**					
	**New medication or dose adjustment**				
		No	57 (75.0)	295 (80.0)	361 (79.2)
		Yes	19 (25.0)	74 (20.1)	95 (20.8)
	**eConsult psychiatry**				
		No	73 (94.8)	343 (92.2)	426 (92.6)
		Yes	4 (5.2)	29 (7.8)	34 (7.4)
	**Referral psychiatry**				
		No	70 (41.7)	324 (75.4)	404 (66.3)
		Yes	98 (58.3)	106 (24.7)	205 (33.7)
	**Referral social worker**				
		No	69 (87.3)	328 (88.4)	408 (88.5)
		Yes	10 (12.7)	43 (11.6)	53 (11.5)
	**Send to ER^a^** **for emergency evaluation**				
		No	76 (100)	369 (100)	456 (100)
		Yes	0 (0)	0 (0)	0 (0)
	**Continue with external specialist**				
		No	60 (79.0)	289 (78.3)	360 (79.0)
		Yes	16 (21.1)	80 (21.7)	96 (21.1)
	**Send to triage RN^b^**				
		No	75 (98.7)	75 (98.7)	449 (98.5)
		Yes	6 (1.6)	1 (1.3)	7 (1.5)
**UHA and SHC (n=1227), n (%)**					
	**C-SSRS^c^ completed**				
		No	276 (33.1)	126 (17.1)	402 (25.5)
		Yes	558 (66.9)	613 (82.9)	1176 (74.5)

a ER: emergency room.

b RN: registered nurse.

c C-SSRS: Columbia-Suicide Severity Rating Scale.

### Patient Characteristics Associated With the Modality of Questionnaire Completion: Asynchronous or Synchronous

Several patient characteristics were associated with asynchronous or synchronous completion of the PHQ-9 (Table 3). The relationship between patient race and completion modality did not meet the level of statistical significance designated by the Bonferroni correction, but the trend is worth noting (*P*=.01). Asian patients were more likely to complete the PHQ-9 asynchronously than White patients (odds ratio [OR] 1.40, 95% CI 1.08-1.82; *P*=.01). Hispanic and Latino patients were less likely than non-Hispanic or non-Latino patients to complete the PHQ-9 asynchronously (OR 0.60, 95% CI 0.45-0.80; *P*<.001). Patient age was associated with modality of PHQ-9 completion, with patients aged 18 to 79 years having a higher likelihood of completing the PHQ-9 asynchronously than patients aged 80 years or older (*P*<.001). Patients with Medicare insurance were less likely (OR 0.64, 95% CI 0.51-0.79; *P*<.001) to complete the PHQ-9 asynchronously than patients with private insurance. Patients with office visits had lower likelihood of completing the PHQ-9 asynchronously than patients with telemedicine visits (OR 0.24, 95% CI 0.20-0.30). Those with moderate to severe depression symptoms were 1.61 times more likely (95% CI 1.21-2.15; *P*=.001) to complete screening asynchronously than those with no or mild symptomatology. Finally, the mean PHQ-9 score (16.4, SD 5.3) for patients who completed the screening asynchronously was significantly higher than that for patients who completed screening synchronously (mean 15.6, SD 5.7; *P*=.004).

**Table 3.** Logistic regression of the association between patient characteristics and whether patients completed the Patient Health Questionnaire-9 (PHQ-9) synchronously or asynchronously (n=1683).

Characteristics		Asynchronous PHQ-9	
		OR^a^ (95% CI)	*P* value
**Sex**			**.30**
	Male	1	
	Female	1.11 (0.91-1.35)	
**Race**			**.01**
	White	1	
	Black or African American	0.72 (0.48-1.07)	.10
	Asian	1.40 (1.08-1.82)	.01
	Other/unknown	1.07 (0.83-1.37)	.60
**Age group (years)**			**.03**
	≥80	1	
	60-79	1.17 (0.78-1.77)	.50
	40-59	1.66 (0.10-2.49)	.02
	18-39	1.84 (1.25-2.72)	.002
	12-17	0.06 (0.01-0.24)	<.001
**Ethnicity**			**<.001**
	Non-Hispanic/non-Latino	1	
	Hispanic/Latino	0.60 (0.45-0.80)	
**Health insurance**			**<.001**
	Private	1	
	Medicare	0.64 (0.51-0.79)	<.001
	Medicaid	1.03 (0.64-1.68)	.90
	Other/unknown	0.55 (0.33-0.93)	.03
**Encounter type**			**<.001**
	Telemedicine	1	
	Office visit	0.24 (0.20-0.30)	
**Appointment status**			**.001**
	Completed	1	
	Cancelled, left, or no-show	5.55 (1.61-19.12)	
**PHQ-9 categories**			**.001**
	Normal or mild (0-10)	1	
	Moderate to severe (≥10)	1.61 (1.21-2.15)	

a OR: odds ratio; reference groups are represented by OR=1 in the table.

### Differential Clinical Response to Suicide Risk by Asynchronous or Synchronous Modalities

The relationship between the timing of PHQ-9 completion and provider clinical response was evaluated (Table 4). Regardless of timing of PHQ-9 completion, there was a relationship between the response to question 9 and referral to psychiatry (*P*=.002). Those who reported suicidal thoughts “more than half the days” (OR 1.62, 95% CI 1.06-2.48; *P*=.03) or “nearly every day” (OR 2.38, 95% CI 1.38-4.12; *P*=.002) had a higher likelihood of receiving a referral to psychiatry than those who endorsed question 9 “several days.” The likelihood of receiving a referral to psychiatry for those who completed a PHQ-9 asynchronously was lower (OR 0.23, 95% CI 0.16-0.34; *P*<.001) than for patients who completed the screening synchronously. In contrast, the likelihood of a patient completing the C-SSRS during the visit was 2.41 times higher (OR 2.41, 95% CI 1.89-3.06; *P*<.001) for patients who completed the PHQ-9 asynchronously than synchronously.

**Table 4.** Logistic regression of the association between completing the Patient Health Questionnaire-9 (PHQ-9) asynchronously or synchronously and clinical response for the Stanford Health Care (SHC; n=456) and University Healthcare Alliance (UHA; n=1227) samples.

Variable		SHC (n=456)												UHA and SHC (n=1683)	
		New medication or dose adjustment		eConsult psychiatry		Referral psychiatry		Referral social worker		Continue with external specialist		Send to triage RN^a^		C-SSRS^b^ completed	
		OR^c^(95% CI)	*P* value	OR(95% CI)	*P* value	OR(95% CI)	*P* value	OR(95% CI)	*P* value	OR(95% CI)	*P* value	OR(95% CI)	*P* value	OR(95% CI)	*P* value
**Timing of PHQ-9 Completion**															
	Synchronous	1	.30	1	.40	1	<.001	1	.80	1	.90	1	.80	1	<.001
	Asynchronous	0.8(0.42-1.34)		1.54 (0.53-4.52)		0.23 (0.16-0.34)		0.90 (0.43-1.89)		1.0 (0.60-1.90)		1.24 (0.15-10.45)		2.41 (1.89-3.06)	
**Response to the 9th Item of the PHQ-9**															
	Several days	1	.80	1	.40	1	.002	1	.80	1	.90	1	.20	1	.07
	More than half the days	0.80 (0.42-1.51)	.50	1.41 (0.58-3.43)	.40	1.62 (1.06-2.48)	.03	1.28 (0.62-2.63)	.50	1.07 (0.59-1.96)	.80	4.5 (0.89-22.70)	.07	1.09 (0.81-1.49)	.60
	Nearly every day	0.98 (0.43-2.22)	.10	2.07 (0.74-5.81)	.20	2.38 (1.38-4.12)	.002	0.94 (0.32-2.81)	.90	1.20 (0.54-2.64)	.70	3.04 (0.31-29.9)	.30	0.68 (0.47-0.97)	.03

a RN: registered nurse.

b C-SSRS: Columbia-Suicide Severity Rating Scale.

c OR: odds ratio.

## Discussion

### Principal Findings

Shifting depression screening from an in-clinic task to a previsit questionnaire led to a dramatic increase in our system’s ability to effectively screen patients for depression without compromising patient safety. The remote delivery of our depression screening and monitoring program saved front line clinical teams time spent administering the PHQs, offered patients a private setting for symptom reporting, and facilitated more intentional clinical responses from providers.

Consistent with previous studies demonstrating that PHQ-9 scores are higher when completed by patients on their own time via a personal device, our patients reported more severe symptoms on asynchronous PHQs compared to in-clinic PHQs [14,15]. In addition, of all patients who completed a PHQ-9, patients with moderate to severe symptoms were more likely to complete the PHQ-9 asynchronously, supporting the idea that patients with severe symptoms may feel more comfortable self-reporting outside of a clinic setting where they are not subject to time pressure or desire to please their provider [31].

Providers were also more likely to conduct a standardized suicide risk assessment when patients completed the PHQ-9 ahead of their visit. Evidence suggests that the ninth item of the PHQ-9 is an insufficient assessment tool for suicide risk and ideation and should be paired with a validated suicide risk assessment instrument [28]. In this study, once a patient completed a PHQ-9 asynchronously and endorsed question 9, the provider was immediately notified via a high-priority alert in the EMR. That previsit notification allowed providers to prepare to address and appropriately prioritize assessing risk of self-harm when agenda-setting for the clinical encounter [32-34]. In contrast, if a provider learned of a high-risk score during a visit, it may be more challenging to prioritize, particularly if that was not a primary reason for the patient presenting to the visit. Mental health concerns can be complex and time-consuming to address. However, these symptoms can also be life-threatening, requiring timely clinical action. Placing a referral to psychiatry is appropriate clinical management of a patient with severe depression. However, when pressed for time or taken by surprise during a visit, and particularly when confronted with severe symptoms, providers may be more likely to delegate further evaluation and management to a psychiatrist. Conversely, when given advance notice of a high-risk score, providers may be better able to prioritize a patient’s mental health concern and take the time to more completely assess, triage, and manage a patient’s depression at the point of care, reducing the need to refer and providing more timely clinical intervention.

Asynchronous PHQ-9 completion offers some potential advantages, such as increased reporting of severe symptoms and supporting more intentional clinical responses, and should be accessible to all patients seen in health systems offering remote screening. Since suicidal risk can fluctuate over time [35], asynchronous screening programs offer an opportunity to evaluate risk in patients on a more frequent basis. Universal depression screening programs, in general, can reduce bias by addressing important barriers, such as care team discomfort and screening based on a provider’s subjective risk assessment [36]. Asynchronous screening can offer a convenient method for vulnerable patient groups with low health care engagement to access resources more directly in primary care [37]. However, technology-enabled programs may disproportionately exclude patients with limited access to digital tools, particularly those with low health or technology literacy [37,38]. We observed that disparities in engagement with PHQ-9 completion existed, despite automation. Specifically, Asian patients were more likely than White patients to complete the PHQ-9 asynchronously, and non-Hispanic or non-Latino patients were more likely to complete screening asynchronously compared to Hispanic or Latino patients. Patients with Medicare insurance were also less likely to complete the PHQ-9 asynchronously, which may be explained by older patients having more of a preference for in-person care than telemedicine [37]. Due to the relative advantages of previsit PHQ-9 completion, health systems may benefit from designing processes to reduce disparities in engagement with electronic tools, such as eCheck-in, and designing in-clinic processes to promote equal opportunities for patients who cannot complete the electronic questionnaire ahead of their visit.

### Limitations

This study was limited in scope and only included data for patients who endorsed question 9 of the PHQ-9 during the program’s first 6 months. Our patient population had a high rate of technology literacy, with approximately 96% of patients enrolled in the web-based patient portal at SHC and 94% of patients enrolled in the web-based patient portal at UHA. Despite widespread engagement with electronic tools, our results suggest health disparities in previsit remote questionnaire completion. Future research should explore whether certain patient groups are more likely to engage in asynchronous questionnaire completion, particularly patients at high risk for depression, and how to bridge this gap.

Another limitation is the lack of clinical response data for UHA patients. Most clinical documentation is available only in free-text portions of providers’ encounter notes, therefore not available discretely in reports and only accessible via manual and detailed chart review, making review of these data on a large scale time prohibitive. Finally, although we believe that no patient attempted or died by suicide in the study population, we only had access to a single data source, the EMR. To increase the likelihood of identifying relevant adverse clinical outcomes related to PHQ-9 completion, during chart review our team placed specific attention on reviewing documentation between the time of questionnaire completion and the patient’s associated clinical encounter. A documented clinical encounter or electronic communication from the patient in the period following questionnaire completion provided confirmation of their living status.

### Conclusions

This study suggests that screening patients for depression outside of a clinical care setting does not compromise patient safety, may increase honest self-reporting for those with severe symptoms, and can allow for more deliberate clinical evaluation and response by providers. Access to asynchronous depression screening may offer essential shifts in patient-centered healthcare access and provider-driven clinical responses. Disparities in patient characteristics associated with asynchronous questionnaire completion present future opportunities to engage non-White and older patients in reporting severe symptoms. Furthermore, this study demonstrates the benefit of exploring processes that support providers’ ability to offer more intentional clinical responses when severe symptoms are identified during clinical encounters. These findings may or may not be limited to the primary care health systems in which the study was conducted. Nonetheless, these conclusions suggest that health systems should consider examining this issue for themselves and potentially reconsider how depression and suicide risk screening are systematically conducted at their institution.

## Abbreviations

C-SSRS: Columbia-Suicide Severity Rating Scale
EMR: electronic medical record
IBH: Integrated Behavioral Health
OR: odds ratio
PHQ-9: Patient Health Questionnaire-9
SHC: Stanford Health Care
SW: social worker
UHA: University Healthcare Alliance
